# The Tarantula Venom Peptide Eo1a Binds to the Domain II S3-S4 Extracellular Loop of Voltage-Gated Sodium Channel Na_V_1.8 to Enhance Activation

**DOI:** 10.3389/fphar.2021.789570

**Published:** 2022-01-14

**Authors:** Jennifer R. Deuis, Lotten Ragnarsson, Samuel D. Robinson, Zoltan Dekan, Lerena Chan, Ai-Hua Jin, Poanna Tran, Kirsten L. McMahon, Shengnan Li, John N. Wood, James J. Cox, Glenn F. King, Volker Herzig, Irina Vetter

**Affiliations:** ^1^ Institute for Molecular Bioscience, The University of Queensland, Brisbane, QLD, Australia; ^2^ Wolfson Institute for Biomedical Research, University College London, London, United Kingdom; ^3^ Australian Research Council Centre of Excellence for Innovations in Peptide and Protein Science, The University of Queensland, Brisbane, QLD, Australia; ^4^ GeneCology Research Centre, University of the Sunshine Coast, Sippy Downs, QLD, Australia; ^5^ School of Science, Technology and Engineering, University of the Sunshine Coast, Sippy Downs, QLD, Australia; ^6^ School of Pharmacy, The University of Queensland, Woolloongabba, QLD, Australia

**Keywords:** voltage-gated sodium channel, Nav1.8, pain, peptide, spider

## Abstract

Venoms from cone snails and arachnids are a rich source of peptide modulators of voltage-gated sodium (Na_V_) channels, however relatively few venom-derived peptides with activity at the mammalian Na_V_1.8 subtype have been isolated. Here, we describe the discovery and functional characterisation of β-theraphotoxin-Eo1a, a peptide from the venom of the Tanzanian black and olive baboon tarantula *Encyocratella olivacea* that modulates Na_V_1.8. Eo1a is a 37-residue peptide that increases Na_V_1.8 peak current (EC_50_ 894 ± 146 nM) and causes a large hyperpolarising shift in both the voltage-dependence of activation (ΔV_50_–20.5 ± 1.2 mV) and steady-state fast inactivation (ΔV_50_–15.5 ± 1.8 mV). At a concentration of 10 μM, Eo1a has varying effects on the peak current and channel gating of Na_V_1.1–Na_V_1.7, although its activity is most pronounced at Na_V_1.8. Investigations into the binding site of Eo1a using Na_V_1.7/Na_V_1.8 chimeras revealed a critical contribution of the DII S3-S4 extracellular loop of Na_V_1.8 to toxin activity. Results from this work may form the basis for future studies that lead to the rational design of spider venom-derived peptides with improved potency and selectivity at Na_V_1.8.

## Introduction

Voltage-gated sodium (Na_V_) channels are pore-forming transmembrane proteins that permit the influx of Na^+^ ions across cell membranes. As such, they are responsible for the rising phase of action potentials and essential for regulating the excitability of neuronal, cardiac and skeletal muscle cells. In humans, there are nine different Na_V_ subtypes (Na_V_1.1–1.9) with distinct physiological functions, attributed to differences in their tissue expression profiles and biophysical properties. Na_V_1.8 is predominantly expressed in the peripheral nervous system, where it is the major contributor to action potential generation and propagation in nociceptive or “pain-sensing” neurons due to its rapid repriming and a depolarised inactivation threshold (Elliott and Elliott, 1993; Akopian et al., 1996; Renganathan et al., 2001; Shields et al., 2012).

Na_V_ channels are large transmembrane proteins, formed by a single polypeptide chain that folds into four homologous (although non-identical) domains (DI–DIV), each consisting of six α-helical transmembrane segments (S1–S6) connected by multiple intracellular and extracellular loops (Catterall et al., 2005). Compared to the other Na_V_ subtypes, the development of selective Na_V_1.8 modulators has been challenging, due to both the scarcity of naturally occurring ligands and the absence of a high-resolution structure to assist in rational drug design. While small molecules with Na_V_1.8 selectivity, such as A-803467 and PF-01247324, have been described (Jarvis et al., 2007; Payne et al., 2015), they are state-dependent inhibitors that bind to DIV preferentially in the inactivated state. As it is unclear how physiologically relevant this state is *in vivo*, more pharmacological tools are required to understand the contribution of Na_V_1.8 to sensory neuron function.

Spider venoms have proven to be a rich source of peptidic Na_V_ modulators, however compared to tetrodotoxin-sensitive isoforms including Na_V_1.7 (Klint et al., 2015; Vetter et al., 2017; Neff and Wickenden, 2021), relatively few spider-venom derived peptides with activity at Na_V_1.8 have been described (Gilchrist and Bosmans, 2012; Cherki et al., 2014; Meng et al., 2016; Peigneur et al., 2018). The aim of this study was to identify novel spider venom-derived peptides with activity at Na_V_1.8. Here, we describe the isolation and pharmacological characterisation of Eo1a, a peptide from the Tanzanian black and olive baboon tarantula *Encyocratella olivacea* that activates Na_V_1.8 by shifting the voltage-dependence of activation to more hyperpolarised potentials.

## Materials and Methods

### Isolation of Eo1a

Venom from *E. olivacea* was extracted via low voltage (9–15 V) electrical stimulations to the basal part of the chelicerae, then lyophilized and stored at –20°C until further use (Guo et al., 2018). Crude *E. olivacea* venom (2 mg dried mass) was dissolved in 5% acetonitrile (ACN)/0.1% trifluoroacetic acid (TFA) and loaded onto an analytical C_18_ reversed-phase (RP) high-performance liquid chromatography (HPLC) column (Kinetex 4.6 × 260 mm, 5 μm; Phenomenex, CA, United States) on a Prominence HPLC system (Shimadzu Scientific Instruments, Rydalmere, NSW, Australia). Venom fractions were collected on the basis of peak at 214 nm eluting at a flow rate of 1 ml/min with solvent A (0.05% TFA in H_2_O) and solvent B (90% ACN, 0.043% TFA in H_2_O) using the gradient: 5% solvent B over 5 min, followed by 5–20% solvent B over 5 min, 20–40% solvent B over 40 min, then 40–80% solvent B over 5 min.

Venom fractions were assessed for activity at hNa_V_1.8 using the FLIPR^TETRA^ membrane potential assay as described previously (Yin et al., 2020). Briefly, venom fractions were incubated for 5 min before activation of Na_V_1.8 by addition of deltamethrin (150 μM). Changes in membrane potential were assessed using the FLIPR^TETRA^ (excitation, 515–545 nm; emission, 565–625 nm) for 25 min and the area under the curve (AUC) after the addition of deltamethrin was computed using ScreenWorks (Molecular Devices, Version 3.2.0.14).

The active fraction was concentrated and further separated by HPLC, and the peptide mass was determined using matrix-assisted laser desorption/ionization time-of-flight (MALDI-TOF) mass spectrometry (MS) using a Model 4700 Proteomics Analyser (Applied Biosystems, Foster City, CA, United States) with α-cyano-4-hydroxycinnamic acid (7 mg/ml in 50% ACN +5% formic acid in H_2_O) as the matrix. The peptide sequence was determined by N-terminal sequencing by the Australian Proteome Analysis Facility (Macquarie University, NSW, Australia).

### Cell Culture

Human Embryonic Kidney (HEK) 293 cells stably expressing human Na_V_1.1 to Na_V_1.7/β1 (SB Drug Discovery, Glasgow, United Kingdom), Chinese Hamster Ovary (CHO) cells stably expressing human Na_V_1.8/β3 in a tetracycline-inducible system (ChanTest, Cleveland, OH, United States), and HEK293 cells stably expressing rat transient receptor potential vanilloid 1 (TRPV1) were cultured as previously described (Yin et al., 2020). Human K_V_2.1 and Na_V_1.7 mutants were transiently transfected into HEK293 cells stably expressing β1/β2 using Lipofectamine 2000 (Thermo Fisher Scientific, Scoresby, VIC, Australia) and used for patch-clamp experiments 48 h after transfection. Cells were grown in an incubator at 37°C with 5% CO_2_ and passaged every 3–4 d (at 70–80% confluency) using TrypLE Express (Thermo Fisher Scientific).

### Na_V_1.7 Channel Mutants

Wild-type (WT) hNa_V_1.7 cDNA (NM_002,977, a gift from Dr James Cox, University College London) was subjected to *in vitro* site-directed mutagenesis using the QuikChange™ XL mutagenesis kit (Agilent Technologies) following the manufacturer’s instructions. Na_V_1.7 mutants were generated by substituting the domain II (DII) S1-S2 loop, the DII S3-S4 loop, the domain IV (DIV) S1-S2 loop and the DIV S3-S4 loop in Na_V_1.7 with the corresponding regions of Na_V_1.8. The mutations were verified by sequencing at the Brisbane node of the Australian Genome Research Facility. Sequence alignments corresponding to the Na_V_1.7 and Na_V_1.8 DII and DIV extracellular loops and the generated chimeras are shown in Supplementary Figure S1.

### Synthesis

Peptides Eo1a and [D24K]Eo1a were assembled by solid-phase peptide synthesis on a Liberty Prime automatic synthesiser (CEM, Matthews, NC, United States) using Fmoc-Rink-amide polystyrene resin at a 0.1 mmol scale. Amino acid sidechain protecting groups were as follows: Asn(Trt), Arg(Pbf), Asp(OMpe), Cys(Trt), Gln(Trt), Glu(OtBu), His(Boc), Lys(Boc), Ser(tBu), Thr(tBu), Trp(Boc) and Tyr(tBu). Fluorenylmethyloxycarbonyl (Fmoc) was removed by 25% pyrrolidine/dimethylformamide (DMF) treatment for 40 s at 105°C. Couplings were performed using 5 eq Fmoc-amino acid/0.25 M Oxyma Pure/2 M *N*,*N′*-diisopropylcarbodiimide in DMF for 1 min at 105°C. Peptides were cleaved from resins and side chains were deprotected using 2.5% triisopropylsilane (TIPS)/2.5% H_2_O/TFA for 2 h. TFA was evaporated under an N_2_ stream, then peptides were washed and precipitated with chilled diethyl ether, dissolved in 50% ACN/0.1% TFA/H_2_O, and lyophilised. Crude peptides were purified by preparative RP-HPLC. Linear peptides were oxidatively folded for 2 days at 4°C in ammonium acetate buffer containing oxidised and reduced glutathione (1:10:100 ratio). The major products were isolated using preparative RP-HPLC, and correct masses were confirmed using electrospray ionization mass spectrometry (ESI-MS).

### Electrophysiology

Automated whole-cell patch-clamp recordings were performed with a QPatch-16 automated electrophysiology platform (Sophion Bioscience, Ballerup, Denmark) using single-hole (QPlate 16 with a standard resistance of 2 ± 0.4 MΩ) or multi-hole (QPlate 16X with a standard resistance 0.2 ± 0.04 MΩ, Na_V_1.8 only) as previously described (McMahon et al., 2020).

The extracellular solution (ECS) consisted of (in mM) 145 NaCl (replaced with 70 choline chloride for Na_V_1.4, Na_V_1.5 and Na_V_1.7), 4 KCl, 2 CaCl_2_, 1 MgCl_2_, 10 HEPES, and 10 glucose, pH to 7.4 with NaOH (adjusted to 305 mOsm/L with sucrose). For Na_V_ recordings the intracellular solution (ICS) consisted of (in mM) 140 CsF, 1 EGTA, 5 CsOH, 10 HEPES, and 10 NaCl, pH to 7.3 with CsOH (adjusted to 320 mOsm/L with sucrose). For K_V_2.1 recordings the ICS consisted of (in mM) 50 KCl, 60 KF, 10 EGTA, 10 HEPES, 10 NaCl pH to 7.3 with KOH (adjusted to 320 mOsm/L with sucrose). TTX (1 μM) was added to the ECS for Na_V_1.8 recordings to inhibit background endogenous TTX sensitive current in CHO cells.

Concentration-response curves at Na_V_1.8 were acquired using a holding potential of −90 mV and a 50 ms pulse to +10 mV every 20 s (0.05 Hz). Eo1a was diluted in ECS with 0.1% bovine serum albumin (BSA) and peptides were incubated with cells for 5 min before measurements were made. Peak current was normalized to buffer control and fitted to a four-parameter Hill equation with variable Hill coefficient.

K_V_2.1 currents were acquired using a holding potential of −80 mV and a 300 ms pulse to +50 mV every 20 s (0.05 Hz). Eo1a was incubated for 5 min and compared to buffer control.

*I-V* curves were obtained with a holding potential of −90 mV followed by a series of 500 ms step pulses that ranged from −110 to +80 mV in 5-mV increments (repetition interval 5  s) before and after 5 min incubation with Eo1a (10 μM). Conductance-voltage (*G*-*V*) curves were obtained by calculating the conductance (*G*) at each voltage (*V*) using equation *G* = *I*/(*V* − *V*_*rev*_), where *V*_*rev*_ is the reversal potential. *G*-*V* curves were fitted with a Boltzmann equation. The voltage dependence of steady-state fast inactivation was examined using a 10-ms pulse of −20 mV (+10 mV for Na_V_1.8) immediately after the 500-ms step above to assess the available non-inactivated channels.

The time constant of fast inactivation (τ) was computed by fitting the current decay traces with a single exponential function using QPatch Assay Software 5.6 (Sophion). The time to peak was calculated from pulse onset to peak inward current using QPatch Assay Software 5.6 (Sophion).

### TRPV1 Calcium Assay

The activity of Eo1a at rTRPV1 was assessed using the assay described previously (Yin et al., 2020). Briefly, the TRPV1 activator capsaicin (300 nM) and Eo1a (10 μM) were added to cells loaded with Calcium 4 no-wash dye (Molecular Devices) and changes in fluorescence were assessed using a FLIPR^TETRA^ (excitation 470–495 nm, emission 515–575 nm) every 1 s for 300 s. Fluorescence responses were normalized to baseline fluorescence (ΔF/F) and plotted versus time using ScreenWorks 3.2.0.14.

### Animals

Adult male C57BL/6J mice aged 4–8 weeks were sourced from the Animal Resource Centre (Canning Vale, Western Australia). Adult male Na_V_1.8 knockouts (Nassar et al., 2004) (aged 8–11 weeks) and wild-type littermates were housed and tested at University College London. Animals were housed in groups of three or four per cage under 12-h light-dark cycles and were provided with standard rodent chow and water *ad libitum*. Ethical approval for experiments involving animals was obtained from The University of Queensland animal ethics committee (TRI/IMB/093/17, IMB/PACE/421/18) and the United Kingdom Home Office (project licence PPL 70/7382). All experiments were conducted in accordance with the *International Association for the Study of Pain Guidelines for the Use of Animals in Research* and the *Australian Code of Practice for the Care and Use of Animals for Scientific Purposes*, 8th edition (2013).

### Calcium Imaging of Dorsal Root Ganglion (DRG) Neurons

For Ca^2+^ imaging experiments, DRG neurons were isolated from 4 week old male C57BL/6J mice as previously described (Robinson et al., 2018). DRG neurons were dissociated and plated in 96-well poly-D-lysine-coated culture plates grown in Dulbecco’s modified Eagle’s medium supplemented with 10% fetal bovine serum (FBS) and penicillin/streptomycin and cultured for 24 h at 37°C with 5% CO_2_. Dissociated DRG neurons were loaded with Fluo-4 AM calcium indicator (5 μM) for 1 h, then washed with Hanks’ balanced salt solution (HBSS) containing 20 mM HEPES. Fluorescence was measured using a Nikon Ti-E Deconvolution inverted microscope, in conjunction with a Lumencor Spectra LED light source (excitation, 485 nm; emission, 521 nm). Images were acquired at 1 frame per s using a 20× objective. For each experiment, baseline fluorescence was observed for 20 s before addition of HBSS +20 mM HEPES containing 0.1% BSA (negative control) at 30 s, 10 μM Eo1a at 60 s, and 30 mM KCl (positive control) (±1 μM TTX) at 200 s. Only excitable cells responding to KCl were included for analysis. Cells were classified as responders if they had at least a 1.5-fold increase in fluorescence over baseline.

### Behavioural Assessment

Eo1a (1 μM or 10 μM) was diluted in saline/0.1% BSA and administered by intraplantar injection to the left hind paw of mice in a volume of 40 μl under isoflurane (3%) anaesthesia, and spontaneous pain behaviours (licks, bites, shakes, and lifts of the hind paw) were counted by a blinded investigator for up to 40 min after the injection.

### Data Analysis

Data were plotted and analyzed using GraphPad Prism version 9.0.0. Statistical significance was defined as *p* < 0.05 using tests as indicated. Data are presented as mean ± SEM unless otherwise stated.

## Results

### Isolation of the Spider Venom Peptide β-TRTX-Eo1a From *Encyocratella olivacea*

**FIGURE 1:**
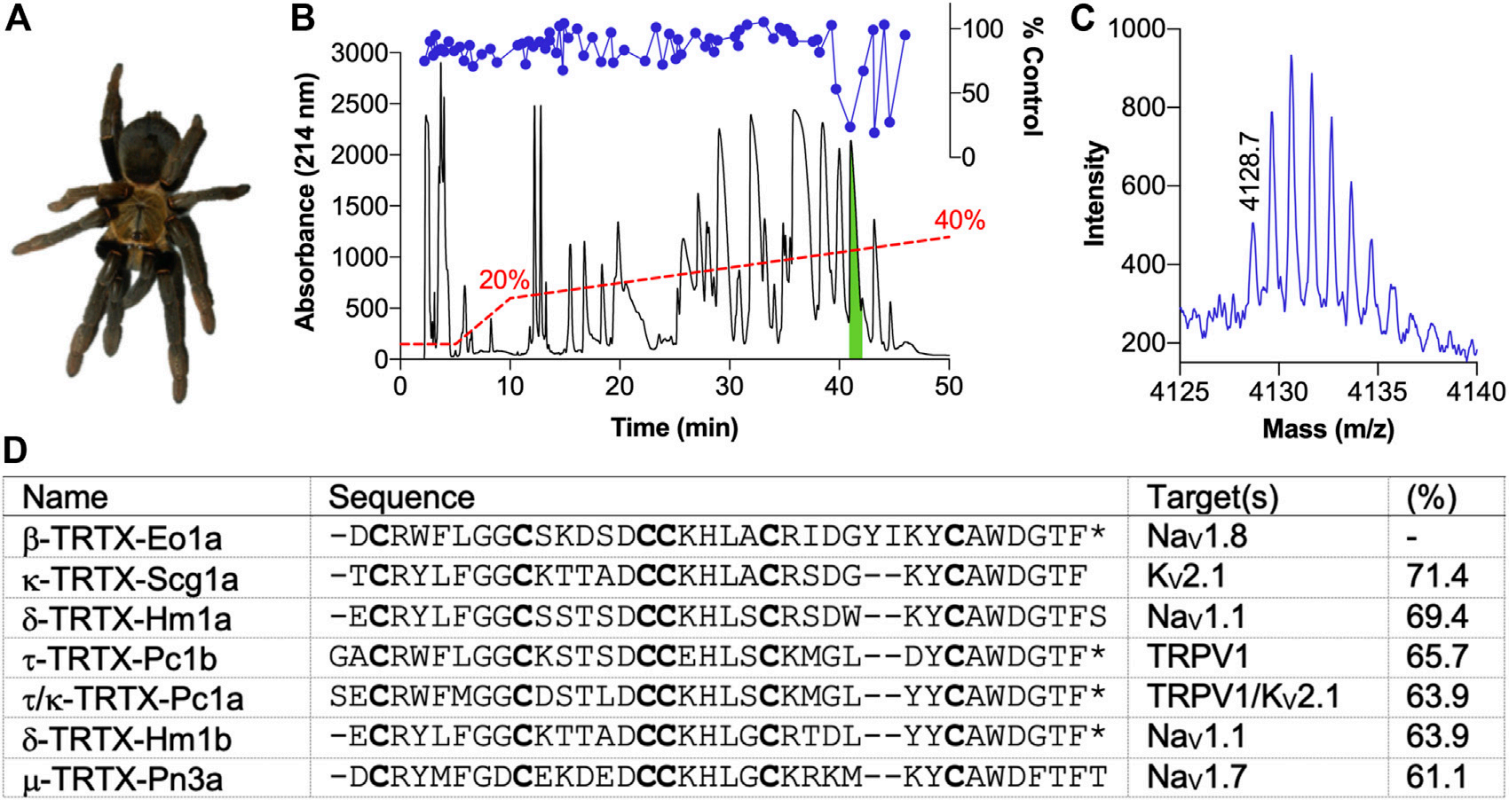
Isolation of the novel peptide β-TRTX-Eo1a from the venom of ***Encyocratella olivacea.* (A)** Photo of a female *E. olivacea* specimen from which crude venom was obtained. **(B)** Chromatogram resulting from fractionation of the crude venom using RP-HPLC (red dashed line indicates acetonitrile gradient). Corresponding activity of each fraction to modulate deltamethrin-induced Na_V_1.8 responses is shown above (blue circles). The green indicates the active peak that was followed up. **(C)** MALDI-TOF MS spectrum showing the M+H^+^ ions for the dominant mass present in the active peak. **(D)** Sequence of Eo1a identified by N-terminal sequencing and alignment to selected spider-venom peptides with a known target from UniProt (www.uniprot.org). Cysteine residues are shown in bold; *, amidated C-terminus. Percentage indicates sequence identity.

Crude venom from *E. olivacea* (Figure 1A) modulated deltamethrin-induced membrane potential changes in CHO cells stably expressing hNa_V_1.8, with activity-guided fractionation isolating this activity to three late-eluting peaks, with the most abundant peak (highlighted in green) chosen for follow up (Figure 1B). The other two peaks likely contained closely related peptides given their similar elution times and activity profiles, so they were not pursued further. MALDI-TOF MS indicated that this peak was dominated by a single (M + H)^+^ of 4,128.7 m/z (Figure 1C). N-terminal sequencing revealed a novel 37-residue peptide that we named β-TRTX-Eo1a (hereafter Eo1a) based on the rational nomenclature for peptide toxins (Figure 1D) (King et al., 2008). The calculated mass and observed mass differed by –1 Da, indicating an amidated C-terminus. Alignment to peptide sequences from the Universal Protein Resource (www.uniprot.org) revealed that Eo1a shares high sequence homology (61–71%) to the K_V_2.1 inhibitor Scg1a (Lee et al., 2004), the Na_V_1.1 activators Hm1a/b (Osteen et al., 2016; Chow et al., 2020), the TRPV1 activators Pc1a/b (Siemens et al., 2006) and the selective Na_V_1.7 inhibitor Pn3a (Deuis et al., 2017). Therefore, we tested the activity of Eo1a at Na_V_1.1 to 1.8, K_V_2.1 and TRPV1. For all further experiments, we used synthetic Eo1a with C-terminal amidation, which co-eluted with the purified native peptide (Supplementary Figure S2), confirming that the synthetic peptide adopts the native disulfide bond configuration.

### Eo1a is an Activator of Na_V_1.8

**FIGURE 2:**
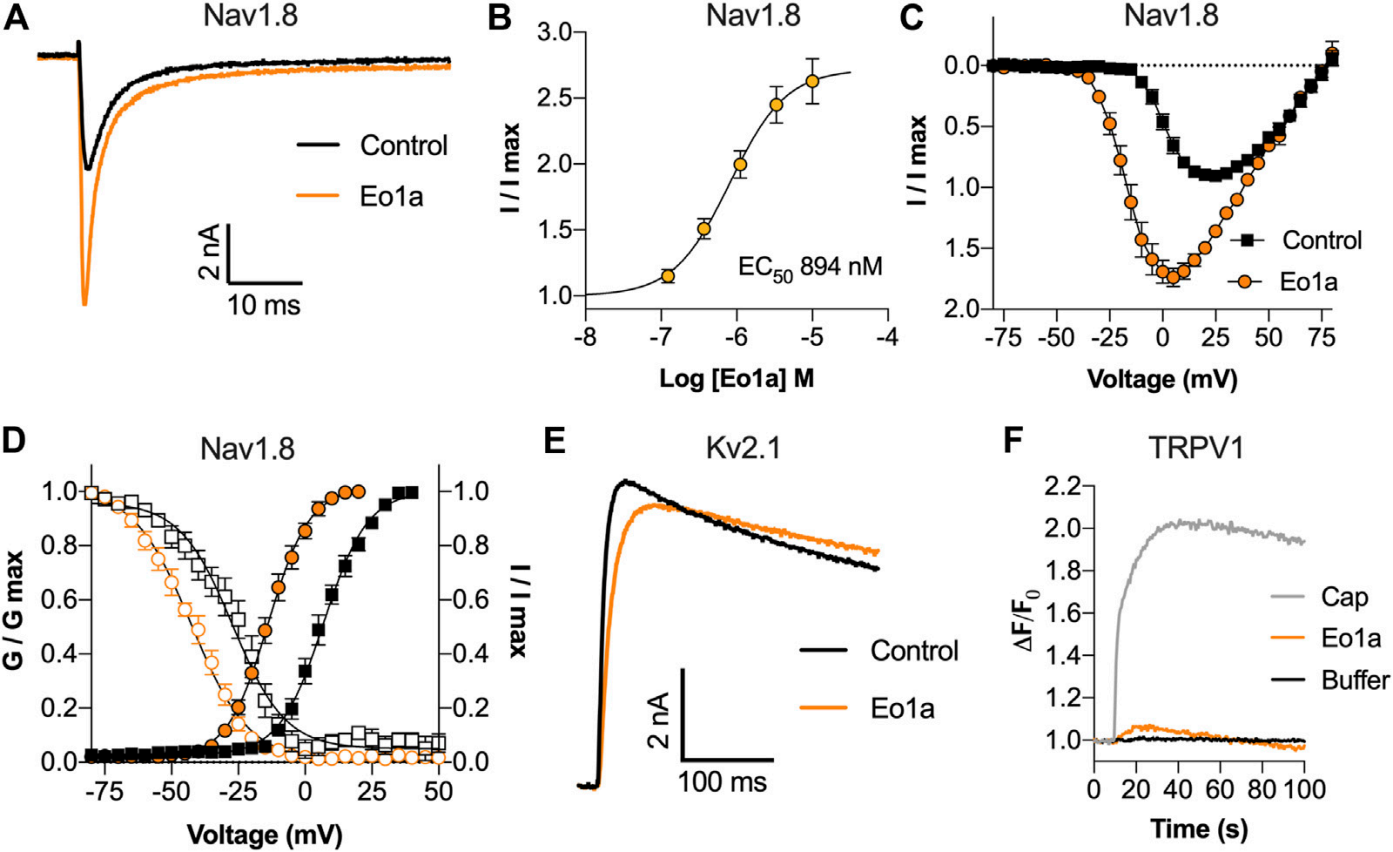
Activity of Eo1a at Na_V_1.8, K_V_2.1 and TRPV1. **(A)** Representative Na_V_1.8 current trace before and after addition of 1 μM Eo1a. Currents were elicited by a 50 ms pulse to +10 mV from a holding potential of −90 mV. **(B)** Eo1a increased Na_V_1.8 peak current with an EC_50_ of 894 ± 146 nM (*n* = 7 cells). **(C)** *I*-*V* relationship before and after addition of 10 μM Eo1a (*n* = 4 cells). **(D)** Conductance-voltage (filled symbols) and steady-state fast inactivation (open symbols) before (black) and after (orange) addition of 10 μM Eo1a (*n* = 4 cells). Eo1a shifted the voltage dependence of activation V_1/2_ by −20.5 mV and inactivation V_1/2_ by −15.5 mV. Data are presented as mean ± SEM. **(E)** Representative K_V_2.1 current trace before and after addition of 10 μM Eo1a. Currents were elicited by a 300 ms pulse to +50 mV from a holding potential of −80 mV. **(F)** Changes in Ca^2+^ dye fluorescence over baseline in TRPV1-expressing cells after addition of capsaicin (300 nM), Eo1a (10 μM) or buffer (mean of *n* = 3 wells).

Functional characterisation of Eo1a by whole-cell patch-clamp electrophysiology revealed that it is a Na_V_1.8 activator. Specifically, Eo1a caused a concentration-dependent increase in peak current with an EC_50_ of 894 ± 146 nM, making it the most potent modulator of Na_V_1.8 described from spider venom to date (Figures 2A,B). We next assessed the effect of Eo1a on the current-voltage relationship at Na_V_1.8, given that spider venom-derived peptides generally bind to the voltage-sensing domains to alter the biophysical properties of Na_V_ channels (Bosmans and Swartz, 2010). Indeed, Eo1a (10 μM) caused a large hyperpolarising shift in the voltage-dependence of activation (ΔV_50_–20.5 ± 1.2 mV) and an increase in total peak current (Figures 2C,D), without significantly altering activation or fast inactivation kinetics of Na_V_1.8 (Supplementary Figure S3). Eo1a (10 μM) also caused a large hyperpolarising shift in the voltage-dependence of steady-state fast inactivation of Na_V_1.8 (ΔV_50_–15.5 ± 1.8 mV), resulting in a comparatively unchanged window current (Figure 2D). Despite being homologous to the K_V_2.1 inhibitor Scg1a, Eo1a (10 μM) had no inhibitory activity on the late current size of K_V_2.1 (I_250 ms_: buffer 6.25 ± 0.9 nA; Eo1a 6.34 ± 0.9 nA; *n* = 3) but did slow the kinetics of activation (τ_activation_: buffer 5.1 ± 0.4 ms, Eo1a 13.6 ± 2.7 ms; *n* = 3) (Figure 2E). Compared to Pc1a/b, which activate TRPV1 alone with the same maximum effect size as capsaicin (Siemens et al., 2006), Eo1a had minimal activity at TRPV1 (Figure 2F).

### Selectivity of Eo1a at Na_V_1.1 to Na_V_1.8

**FIGURE 3:**
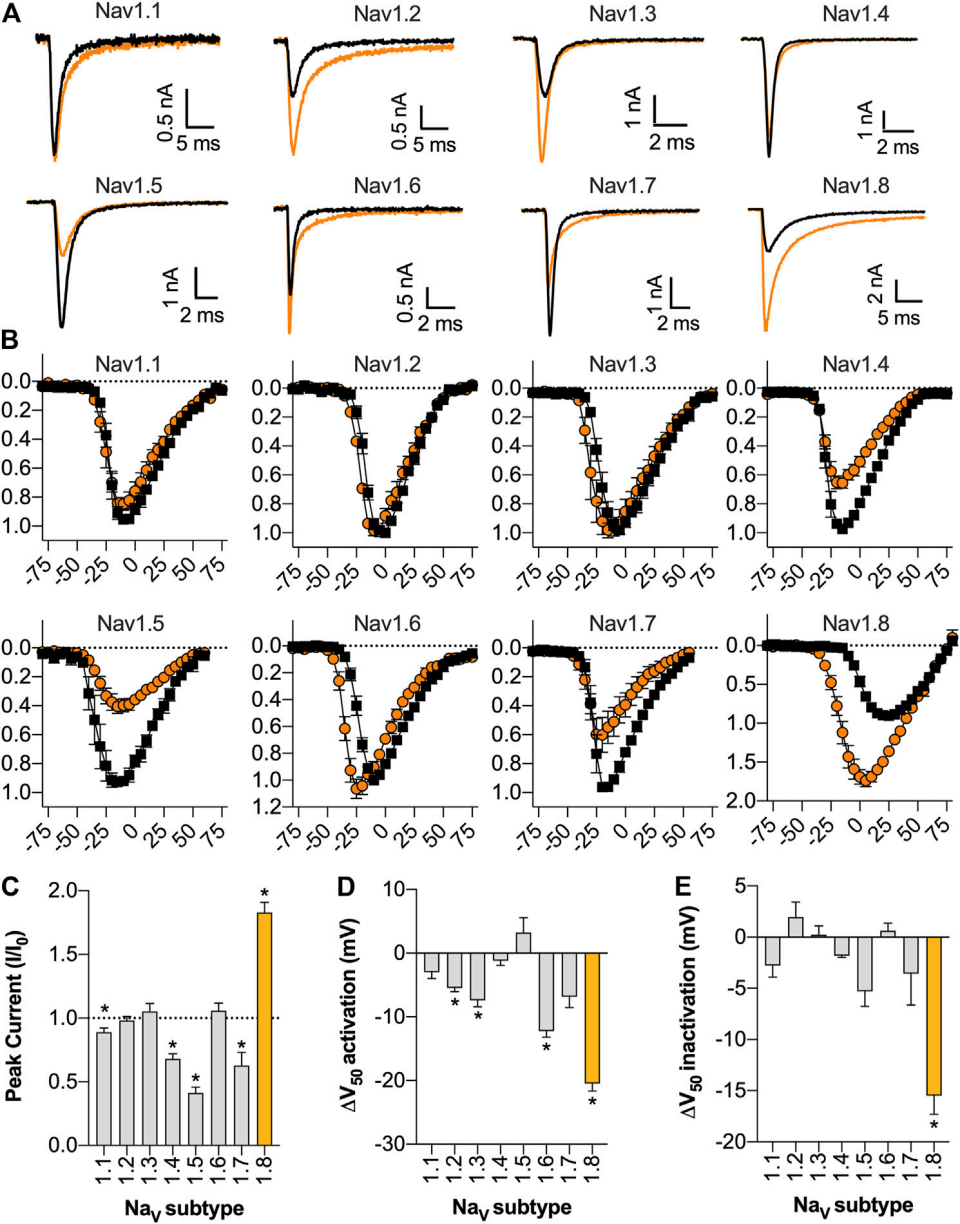
Selectivity of Eo1a at Na_V_1.1 to Na_V_1.8. **(A)** Representative Na_V_1.1 to 1.8 current traces before (black) and after addition of 10 μM Eo1a (orange). Currents were elicited by a 50 ms pulse to −20 mV (for Na_V_1.1 to Na_V_1.7) or to +10 mV (for Na_V_1.8) from a holding potential of −90 mV. **(B)** *I*-*V* relationship before and after addition of 10 μM Eo1a at Na_V_1.1 to Na_V_1.8. Na_V_1.8 panel is same as presented in Figure 2C but included again here for comparison. y-axes represent normalised current (*I*/*I*_0_) and x-axes represent membrane potential (mV). **(C)** Peak current taken from the *I*-*V* protocol (at any voltage) after addition of 10 μM Eo1a normalised to buffer control. Statistical significance was determined using one sample *t*-test compared to hypothetical mean of 1, *p < 0.05. **(D)** Change in the V_1/2_ of activation or **(E)** V_1/2_ of steady-state fast inactivation after addition of 10 μM Eo1a. Statistical significance was determined using paired *t*-tests with Holm-Sidak’s multiple comparisons test compared to buffer control, *p < 0.05 (see Table 1). Data are presented as mean ± SEM (*n* = 4–6 cells).

Given the high sequence homology between Na_V_ subtypes, we next assessed the activity of Eo1a at Na_V_1.1 to Na_V_1.7. At a test pulse of −20 mV, Eo1a (10 μM) had minimal effect on the inactivation kinetics of Na_V_1.1 to Na_V_1.5, but delayed fast-inactivation of Na_V_1.6 and Na_V_1.7 (Figure 3A). When comparing the peak current from the current-voltage protocols (which accounts for shifts in the peak due to changes in the V_1/2_ of activation), Eo1a increased Na_V_1.8 peak current (*I*/*I*_0_ = 1.8 ± 0.08), but decreased the peak current of Na_V_1.1 (*I*/*I*_0_ = 0.89 ± 0.03), Na_V_1.4 (*I/I*_0_ = 0.68 ± 0.04), Na_V_1.5 (*I*/*I*_0_ = 0.41 ± 0.05) and Na_V_1.7 (*I*/*I*_0_ = 0.63 ± 0.10) (Figures 3B,C). Eo1a caused a hyperpolarizing shift in the V_1/2_ of activation of Na_V_1.2, Na_V_1.3, and Na_V_1.6, albeit less pronounced than at Na_V_1.8, but it caused no shift in the V_1/2_ of inactivation of Na_V_1.1 to 1.7 (Figures 3D,E; Table 1, Supplementary Figure S4). Of the Na_V_ subtypes expressed in the peripheral nervous system, Eo1a had the most pronounced effects on Na_V_1.8, while also displaying effects consistent with enhanced activity of Na_V_1.6 and Na_V_1.7. Activity at Na_V_1.9 was not tested due to difficulties with heterologous expression of this subtype.

**TABLE 1:** Effects of Eo1a (10 μM) on Na_V_ channel voltage-dependence of activation and steady-state fast inactivation. Data are reported as mean ± SEM (*n* = 4–6). *, *p* < 0.05, paired *t*-tests with Holm-Sidak’s multiple comparisons test compared to buffer control.

	V_1/2_ activation		V_1/2_ inactivation	
	Control	Eo1a	Control	Eo1a
Na_V_1.1	−21.5 ± 1.5 mV	−24.4 ± 2.1 mV	−55.0 ± 1.1 mV	−57.8 ± 2.0
Na_V_1.2	−15.9 ± 1.2 mV	−21.3 ± 0.9 mV*	−54.6 ± 1.6 mV	−52.7 ± 0.5
Na_V_1.3	−20.4 ± 2.2 mV	−27.9 ± 3.0 mV*	−63.2 ± 1.0 mV	−63.0 ± 1.5
Na_V_1.4	−27.6 ± 1.4 mV	−28.8 ± 1.2 mV	−65.8 ± 1.3 mV	−67.6 ± 1.2
Na_V_1.5	−30.3 ± 4.0 mV	−27.1 ± 2.2 mV	−70.2 ± 0.9 mV	−75.5 ± 1.0
Na_V_1.6	−22.7 ± 1.0 mV	−35.0 ± 1.2 mV*	−56.3 ± 1.1 mV	−56.2 ± 1.0
Na_V_1.7	−26.1 ± 1.5 mV	−32.9 ± 2.9 mV	−65.5 ± 1.4 mV	−69.1 ± 4.3
Na_V_1.8	+7.0 ± 1.8 mV	−13.5 ± 1.6 mV*	−27.4 ± 2.7 mV	−42.9 ± 2.1*

### Eo1a Activates Sensory Neurons and Causes Spontaneous Pain Behaviours *in vivo*

**FIGURE 4:**
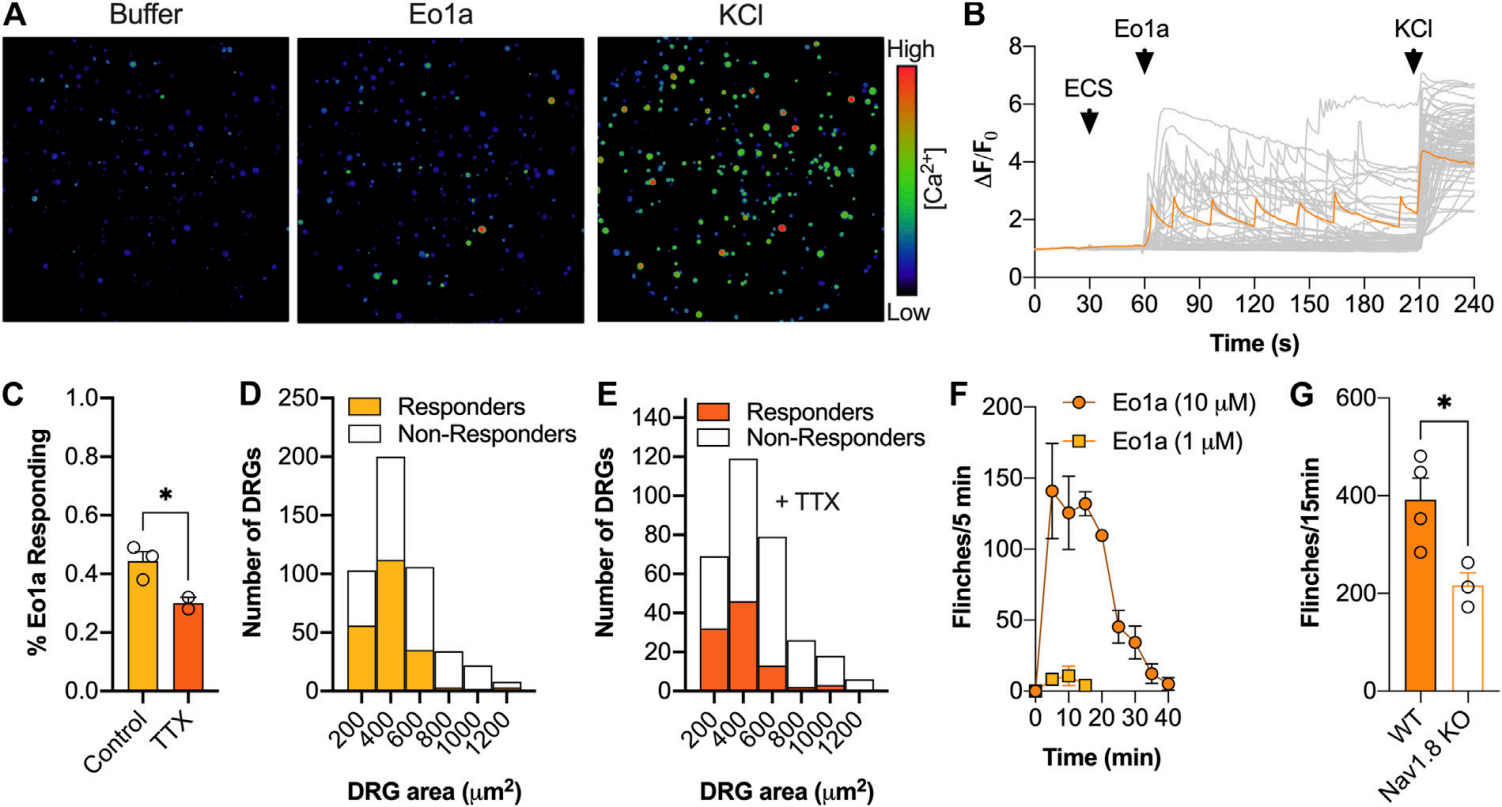
Effect of Eo1a on peripheral sensory neurons. **(A)** Pseudocolor image illustrating Ca^2+^ responses in DRG neurons after addition of buffer, Eo1a (10 μM) and KCl (30 mM) with **(B)** corresponding traces from all neurons of one representative experiment (*n* = 1 well). One representative trace of a neuron activated by Eo1a is highlighted in orange. **(C)** Proportion of neurons activated by Eo1a (10 μM) ± TTX (1 μM). Data is presented as mean ± SEM of 2–3 independent wells. **(D)** Size distribution of neurons activated by Eo1a alone (*n* = 473) or **(E)** in the presence of Eo1a and TTX (*n* = 317). **(F)** Intraplantar administration of Eo1a (1 μM, 10 μM) induced spontaneous pain behaviours in mice **(G)** that were significantly reduced in Na_V_1.8 knockout mice compared to WT littermate controls. Data are presented as mean ± SEM (*n* = 3–4 mice). Statistical significance was determined using unpaired *t*-test, *p < 0.05.

Because of its relative selectivity for Na_V_1.8, we next assessed the effect of Eo1a on primary sensory neurons and pain behaviours *in vivo*. Application of Eo1a (10 μM) to dissociated DRG neurons caused Ca^2+^ influx in 44 ± 3% of neurons, consistent with Na_V_ activator activity (Figures 4A–C). In the presence of TTX (1 μM), the percentage of neurons activated by Eo1a was reduced to 30 ± 2%, suggesting most of the activity is mediated via activation of Na_V_1.8, although activation of the TTX-sensitive channels Na_V_1.6 and Na_V_1.7 may also contribute. In line with the known expression profile of Na_V_1.8 (Shields et al., 2012), neurons activated by Eo1a were of smaller size, and the size distribution did not change in the presence of TTX (Figures 4D,E). When administered by intraplantar injection in mice, Eo1a (10 μM) caused spontaneous pain behaviours that gradually subsided over 30 min, consistent with Na_V_1.8 activation (Figure 4F). Consistent with the activity observed in DRG neurons, Eo1a-induced spontaneous pain was significantly reduced, but not abolished in Na_V_1.8 knockout mice, suggesting that activity at other subtypes likely contributes to its nociceptive activity *in vivo* (Flinches/15 min: WT mice 392 ± 45; Na_V_1.8 KO mice 216 ± 26 (Figure 4G).

### Eo1a Binds to the DII S3-S4 Extracellular Loop of Na_V_1.8

**FIGURE 5:**
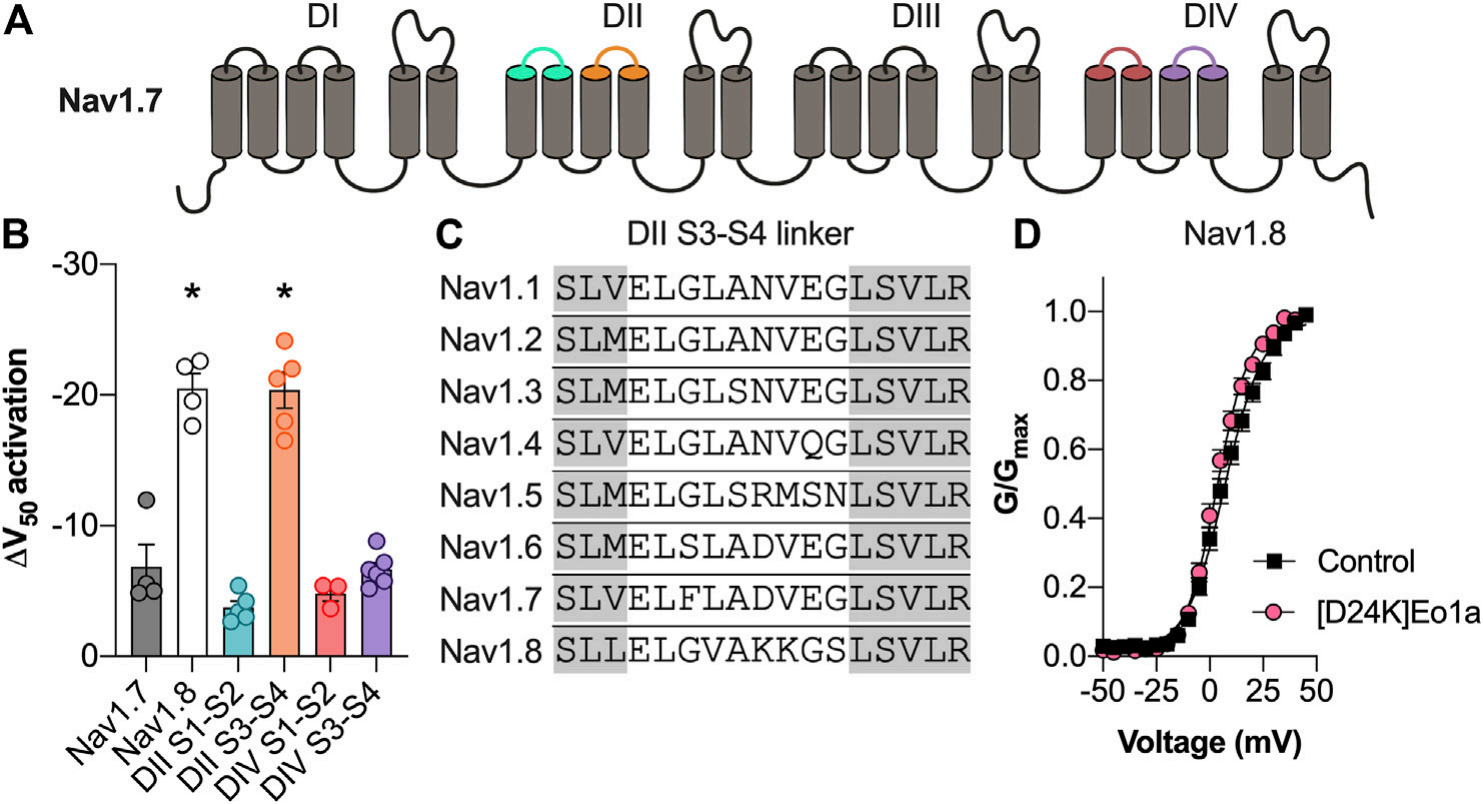
Activity of Eo1a at Na_V_1.7/Na_V_1.8 channel mutants. **(A)** Schematic representation of the Na_V_1.7 extracellular loops replaced with the corresponding regions of Na_V_1.8. **(B)** Change in the V_1/2_ of activation after application of Eo1a (10 μM) at Na_V_1.7 where the DII S1-S2, DII S3-S4, DIV S1-S2 and DIV S3-S4 extracellular loops have been replaced by the corresponding extracellular loops of Na_V_1.8 (*n* = 3–6 cells). Eo1a binds to the DII S3-S4 loop of Na_V_1.8 to shift the voltage-dependence of activation. Statistical significance was determined using one-way ANOVA with Dunnett’s multiple comparisons test compared to wildtype Na_V_1.7, *p < 0.05 **(C)** Sequence alignment of the DII S3-S4 extracellular loop of human Na_V_1.1 to Na_V_1.8. Grey shading indicates the transmembrane regions. **(D)** Conductance-voltage relationship of [D24K] Eo1a (10 μM) on Na_V_1.8 (*n* = 8 cells). Data are presented as mean ± SEM.

To determine the binding site of Eo1a, we capitalised on its relative selectivity for Na_V_1.8 over Na_V_1.7 and generated Na_V_1.7 channel mutants in which the extracellular loops of DII S1-S2, DII S3-S4, DIV S1-S2 and DIV S3-S4 were replaced with the corresponding regions of Na_V_1.8 (Figure 5A).

Only insertion of the Na_V_1.8 DII S3-S4 extracellular loop into Na_V_1.7 could recapitulate the large hyperpolarising shift in the voltage-dependence of activation of Eo1a (10 μM) at native Na_V_1.8 (ΔV_1/2_ activation: DII S1-S2 −3.7 ± 0.5 mV; DII S3-S4 −20.4 ± 1.4 mV; DIV S1-S2 −4.8 ± 0.6 mV; DIV S3-S4 −6.7 ± 0.5 mV) indicating that the Na_V_1.8 DII S3-S4 extracellular loop is the primary binding site for Eo1a (Figure 5B).

Although the equivalent loss-of-function mutation in Na_V_1.8 could unfortunately not be characterised due to difficulties with the expression, we further confirmed interaction of Eo1a with DII S3-S4 of Na_V_1.8 by rational peptide analogue design. Given that K24 of the related Na_V_1.7 inhibitor Pn3a is modelled to interact with the DII S3-S4 extracellular loop of Na_V_1.7 (Mueller et al., 2020), we hypothesised that the negatively charged D24 at the equivalent position of Eo1a would be important for activity. Indeed, replacement of the negatively charged D24 on Eo1a with a lysine caused loss of activity at Na_V_1.8, with [D24K]Eo1a no longer able to cause the same shift in the voltage-dependence of activation (ΔV_1/2_ activation −3.8 ± 0.8 mV) (Figure 5D).

## Discussion

Here we describe the isolation and pharmacological characterisation of the spider venom-derived peptide Eo1a. Eo1a is an activator of Na_V_1.8, causing an increase in peak current without altering the activation or fast inactivation kinetics. Interestingly, the increase in peak current is not entirely accounted for by the hyperpolarizing shift in the voltage-dependence of activation. The macroscopic current is a function of the total number of channels, the single channel current, and the open probability (Sigworth, 1980), and therefore if we assume that the former two parameters remain constant, it is most likely that Eo1a increases the open probability of Na_V_1.8, although this remains to be experimentally confirmed by single-channel recordings.

To our knowledge, Eo1a is the most selective activator of Na_V_1.8 described to date; nevertheless, our data indicates that Eo1a has some off-target activity, which limits its use as a pharmacological tool to selectively activate Na_V_1.8 in native neurons. Indeed, spider-venom peptides related to Eo1a (belonging to NaSpTx family 2) are known to have promiscuous activity at Na_V_, K_V_, and Ca_V_ channels (Klint et al., 2012), and the activity of Eo1a at other K_V_ subtypes and Ca_V_ channels remains to be assessed. Nevertheless, Eo1a is a useful tool to probe the structure-function relationships of Na_V_1.8 in heterologous expression systems. In order to improve the potency and/or selectivity of Eo1a for Na_V_1.8, further structure-activity relationship studies would need to be undertaken. Compared to the other related spider venom-derived peptides, Eo1a has two additional residues in loop 4, and amino acids in this loop have previously been shown to contribute to the pharmacophore of this family (Wang et al., 2004; Mueller et al., 2020; Yin et al., 2020). In line with this, we have shown that the negatively charged aspartic acid at position 24 in Eo1a is crucial for activity. Therefore, structure-activity studies focused on loop 4 would likely provide additional insights into the pharmacophore of Eo1a and may facilitate the rational design of Na_V_1.8-selective analogues.

Our data on the Na_V_1.7/Na_V_1.8 extracellular loop chimeras indicate that Eo1a binds to the DII S3-S4 loop of Na_V_1.8 to cause a hyperpolarizing shift in the voltage-dependence of activation. Sequence alignment of the DII S3-S4 extracellular loop of Na_V_1.7 and Na_V_1.8 highlights a major difference in charged amino acid residues, namely D816 and E818 in Na_V_1.7 and K816 and K817 in Na_V_1.8, which likely account for differences in Eo1a activity between the two subtypes (Figure 5C). Given that K24 of the related Na_V_1.7 inhibitor Pn3a (Figure 1D) is modelled to interact with E818 of Na_V_1.7 (Mueller et al., 2020), and Eo1a has a negatively charged D24 at the equivalent position, we hypothesised that this residue would be important for activity. Indeed, as [D24K]Eo1a was no longer able to shift the voltage-dependence of activation, this suggests that D24 on Eo1a interacts with positively charged residues on the Na_V_1.8 DII S3-S4 extracellular loop. While the DII S3-S4 extracellular loop is the common binding site for many spider-venom derived peptides with potent inhibitory activity at Na_V_1.7 (Xiao et al., 2008; Deuis et al., 2017; Shen et al., 2019; Wisedchaisri et al., 2021), this, to our knowledge, is the first spider-venom derived peptide shown to interact with the DII S3-S4 extracellular loop of Na_V_1.8.

The effect of Eo1a on the biophysics of the other Na_V_ subtypes is quite variable. For example, Eo1a causes a hyperpolarising shift in the voltage-dependence of activation at Na_V_1.2, Na_V_1.3 and Na_V_1.6, but inhibits peak current at Na_V_1.5 without shifting the voltage-dependence of activation. Interestingly, a similar activity profile is reported for the β-scorpion Ts1, which presumably also interacts with the DII S3-S4 extracellular loop (Peigneur et al., 2015). Therefore, it is likely Eo1a interacts with the DII S3-S4 extracellular loop at Na_V_1.1-Na_V_1.7 to exert its pharmacological effects. However, the toxin-channel interactions that give rise to these different biophysical effects at different Na_V_ subtypes remain to be determined.

In conclusion, we have identified a novel spider-venom peptide that modulates Na_V_1.8 gating by binding to the DII S3-S4 loop. Our results provide the basis for further structure-activity relationship studies to rationally design spider-venom peptides with improved activity at Na_V_1.8, which may be used as pharmacological tools or analgesic drug leads.

## Data Availability

The original contributions presented in the study are included in the article/Supplementary Material, further inquiries can be directed to the corresponding author.
