# Supplementary material for: The Tarantula Venom Peptide Eo1a Binds to the Domain II S3-S4 Extracellular Loop of Voltage-Gated Sodium Channel NaV1.8 to Enhance Activation
Source: Front Pharmacol. 2022 Jan 14;12:789570. doi: 10.3389/fphar.2021.789570 (PMC8795738; doi:10.3389/fphar.2021.789570)
Supplement: Supplementary file 1 [file DataSheet1.docx]

Supplementary Material

# Supplementary Figures

**Supplementary Figure 1.** Sequence alignments corresponding to the Na_V_1.7 and Na_V_1.8 DII and DIV extracellular loops and the generated chimeras used in this study.

**Supplementary Figure 2.** Chromatogram of native Eo1a purified from crude venom overlaid with synthetic Eo1a run on a Shimadzu LC-40D XR system using a Thermo Hypersil GOLD 2.1 x 100 mm C18 column heated at 40°C with flow rate of 0.3 mL/min using the following gradient: 0 to 50% B over 50 min (solvent A, 0.05% TFA in H_2_O; solvent B, 90% ACN, 0.05% TFA in H_2_O).

**Supplementary Figure 3.** Eo1a has minimal effect on the activation and fast inactivation kinetics of Na_V_1.8. **(A)** Fast inactivation time constants as determined by a single exponential fit at potentials between −5 mV and +45 mV. **(B)** Time to peak calculated from pulse onset to peak inward current at potentials between −5 mV and +45 mV. Data are presented as mean ± SEM (n = 4 cells). Statistical significance was tested using two-way ANOVA, **P* < 0.05.

**Supplementary Figure 4.** The conductance-voltage (squares; left axis) and steady-state fast inactivation (circles; right axis) curves at Na_V_1.1-Na_V_1.7 before (black) and after addition of Eo1a 10 μM (orange). Data are presented as mean ± SEM (n = 4-6 cells).
